# Treatment of Severe COVID-19 with Tocilizumab Mitigates Cytokine Storm and Averts Mechanical Ventilation during Acute Respiratory Distress: A Case Report and Literature Review

**DOI:** 10.3390/tropicalmed5030112

**Published:** 2020-07-03

**Authors:** Faryal Farooqi, Naveen Dhawan, Richard Morgan, John Dinh, Kester Nedd, George Yatzkan

**Affiliations:** 1 Department of Internal Medicine, Larkin Community Hospital, South Miami, FL 33143, USA; naveendhawan@hotmail.com (N.D.); morgan@larkinhospital.com (R.M.); Jdinh@larkinhospital.com (J.D.); knedd01@gmail.com (K.N.); 2 Department of Pulmonary Medicine, Larkin Community Hospital, South Miami, FL 33143, USA; georgeyatzkan@yahoo.com

**Keywords:** COVID-19, SARS-CoV-2, IL-6 inhibitors, tocilizumab, cytokine release syndrome, cytokine storm

## Abstract

COVID-19, caused by the novel severe acute respiratory coronavirus 2 (SARS-CoV-2), emerged in Wuhan, China, in 2019 and has resulted in the current pandemic. The disease continues to pose a major therapeutic challenge. Patient mortality is ultimately caused by acute respiratory distress syndrome (ARDS). Cytokine release syndrome (or “cytokine storm”) is likely to be a contributing factor to ARDS in many patients. Because interleukin 6 (IL-6) is known to play a key role in inflammation, IL-6 receptor inhibitors such as tocilizumab may potentially treat COVID-19 by attenuating cytokine release. We present the case of a 48-year-old male with severe COVID-19, on the verge of meeting intubation requirements, who needed progressive oxygen support for respiratory distress. The patient was treated with a non-weight-based dosage of tocilizumab to prevent the onset of a cytokine storm. We chose to administer an IL-6 inhibitor because of the gradually increasing levels of acute phase reactants identified on serial blood draws, as well as his declining respiratory status. The treatment was well-tolerated in conjunction with standard drug therapies for COVID-19 (hydroxychloroquine, azithromycin, and zinc). The patient subsequently experienced marked improvements in his respiratory symptoms and overall clinical status over the following days. We believe that tocilizumab played a substantial role in his ability to avert clinical decline, particularly the need for mechanical ventilation. Ultimately, the patient was downgraded from the ICU and discharged within days. We highlight the potential of IL-6 inhibitors to prevent the progression of respiratory disease to a point requiring ventilator support. This case underscores the potential importance of early serial measurements of IL-6 and cytokine storm-associated acute phase reactants, such as ferritin, D-dimer, and C-reactive protein, in guiding clinical decision-making in the management of patients with suspected COVID-19. **Conclusion:** The early, proactive identification of serum acute phase reactants should be implemented in the treatment of COVID-19 in order to screen for a primary contributor to mortality—the cytokine storm. This screening, when followed by aggressive early treatment for cytokine storm, may have optimal therapeutic benefits and obviate the need for mechanical ventilation, thereby decreasing mortality. Additionally, we review current evidence regarding cytokine release syndrome in COVID-19 and the use of IL-6 receptor inhibition as a therapeutic strategy, and examine other reported cases in the literature describing IL-6 antagonist treatment for patients with COVID-19.

## 1. Introduction

The novel coronavirus disease 2019 (COVID-19) outbreak started in December 2019 in Wuhan, China, and has emerged as a major pandemic [1,2]. Severe acute respiratory syndrome coronavirus (SARS-CoV-2), an enveloped positive-stranded RNA virus, was later identified as the causative agent [3,4]. As of April 28, 2020, there were more than 3,000,000 reported cases and 200,00 deaths from COVID-19 worldwide [5]. The case-fatality rate of COVID-19 has been estimated to be 2–3%, although estimates vary [6]. Patients with severe cases develop pneumonia that can lead to acute respiratory distress syndrome (ARDS) [3]. Respiratory failure secondary to ARDS in patients with COVID-19 is the most common cause of death [7]. 

Currently, no specific effective drug treatment or vaccine is available for COVID-19 [8,9]. Therapeutic management is supportive, but some repurposed off-label anti-HIV and anti-viral medications are currently in use, including hydroxychloroquine, remdesevir, lopinavir/ritonavir, and interleukin 6 (IL-6) receptor inhibitors, in addition to convalescent plasma therapy [9,10,11,12]. Although several trials are underway, the use of these drugs remains to be substantiated by large, randomized clinical studies; to date, they have only shown promise in anecdotal experiences and circumstantial evidence mostly derived from studies conducted in vitro or in patients in single-arm studies with limited sample sizes and nonrandomized subject populations, which have yielded mixed results [10,13,14,15,16,17,18].

A major clinical feature of COVID-19 is lung-centric pathology resulting in respiratory deterioration, and the most common cause of death is acute respiratory failure due to ARDS [3,19]. According to current data, only 5% of all COVID-19 infections result in ARDS requiring mechanical ventilation, because most infected individuals experience complete recovery [20]. However, 25% of all patients with COVID-19 are believed to clinically progress and acquire critical complications, including ARDS, in which patients may quickly deteriorate and succumb to respiratory failure [21]. In particular, the survival rate among patients who require ventilator support remains poor. In a recent study on ICU patients with COVID-19 in Wuhan, China, only 21% of patients requiring non-invasive mechanical ventilation and 14% of patients requiring invasive mechanical ventilation survived [22]. Therefore, the early management of respiratory symptoms to prevent progression to ARDS and avert the need for mechanical ventilation is critical for preventing mortality. 

Cytokine storm, a hyperinflammatory state mediated by the release of cytokines, is known to be a key cause of ARDS [21]. In this regard, disrupting cytokine storm is an important potential therapeutic approach [21]. Interleukin 6 (IL-6), a multifunctional mediator of inflammation, is widely believed to play a pivotal role in the development of cytokine storm and to eventually cause the ARDS and interstitial pneumonia seen in severe COVID-19 [7,20,23,24]. The attenuation of IL-6 through receptor blockade has been hypothesized to blunt the cytokine storm responsible for respiratory disease progression [20]. 

Promising results of a recent single-arm trial of 21 patients with severe COVID-19 in China in February 2020 showed clinical improvements after therapy with the IL-6 receptor antagonist tocilizumab. Consequently, the Chinese National Health Commission included tocilizumab in the 7th edition of its guide for COVID-19 diagnosis and treatment [20,21,25]. Italian guidelines followed suit and began to suggest the use of tocilizumab in cases of rapid radiologic and clinical deterioration [26,27]. Other trials are currently underway worldwide to better understand the potential therapeutic effects of IL-6 receptor inhibitors for COVID-19 treatment [28,29]. Currently, 23 trials are investigating the use of tocilizumab worldwide [28]. Additionally, ten trials are underway to investigate the role of the IL-6 receptor inhibitor sarilumab for treating COVID-19 [29]. 

Here, we present the case of a patient with severe COVID-19 who was administered tocilizumab, combined with generally accepted therapy at the time (hydroxychloroquine, azithromycin, and zinc), in an ICU setting. From a clinical standpoint, cytokine storm development was observed, as the patient required incremental increases in oxygen therapy (L/min) just prior to tocilizumab administration. He was on the verge of requiring intubation and mechanical ventilation, on the basis of his apparent respiratory distress and arterial blood gas measurements. Interestingly, the patient showed immediate improvement and did not require ventilatory support after he received the IL-6 inhibitor. He eventually recovered and was sufficiently stable for hospital discharge shortly thereafter. The present case highlights the importance of addressing a mounting cytokine storm in its early stages. Our findings imply that inhibition of the IL-6 receptor has profound benefits under certain circumstances of severe COVID-19. 

## 2. Case Presentation

A 48-year-old male cruise ship worker with no significant past medical history presented to our hospital with shortness of breath and the production of sputum. He had reportedly tested positive for COVID-19 before arrival at our hospital. He described having felt sick for seven days while on a cruise ship, with an initial acute onset of symptoms that developed over the course of a day and rapid progression to dyspnea on exertion. He also reported a productive cough with yellow sputum, fever, occasional hemoptysis, nasal congestion, a sore throat, and concomitant nausea and vomiting during the few days before admission. The patient denied experiencing any similar previous episodes. 

On hospital admission, the patient was hypoxic in the emergency department and was placed on a non-rebreather mask. Arterial blood gas analysis after admission revealed a pH of 7.44, PCO_2_ of 34, PO_2_ of 113, and SaO_2_ of 98.2% (with a 15% non-rebreather mask). When the non-rebreather mask was momentarily removed, desaturation occurred, and the SaO_2_ decreased to 88%. 

A chest X-ray revealed infiltrates visible as bilateral hazy opacities (in the right lung more than the left lung), thus suggesting possible multifocal pneumonia or pulmonary edema due to ARDS, along with cardiomegaly. Laboratory results revealed elevated acute phase reactants (erythrocyte sedimentation rate (ESR) 50, C-reactive protein (CRP) > 16, ferritin 672, and lactate dehydrogenase (LDH) 460) and lymphopenia (lymphocyte count 1.2). Additionally, the blood laboratory results showed transaminitis, with elevated aspartate aminotransferase (AST) at 58 and normal alanine aminotransferase (ALT) at 34.

The increased levels of acute phase reactants, an inflammatory biomarker profile elevated from baseline, and lymphopenia supported our suspicion of cytokine storm as a feature of COVID-19 [30]. The broader differential included acute hypoxic respiratory failure and early onset multifocal viral pneumonia with impending ARDS. Importantly, some of the primary acute phase reactants believed to be associated with cytokine storm include ferritin, D-dimer, CRP, troponin, and LDH [31,32,33]. The clinical values of ferritin, D-dimer, and CRP, in addition to the ESR, which is a general marker of inflammation, were closely monitored on a regular basis. Lymphocyte counts were regularly assessed as lymphopenia, which is also believed to be associated with cytokine storm in COVID-19 [31,32,33]. Oxygen saturation was also carefully monitored each day. To confirm SARS-CoV-2 infection, we conducted a nasopharyngeal swab test. The real-time reverse-transcriptase polymerase chain reaction (RT-PCR) assay results later revealed a positive result on day 5.

The treatment included high-flow oxygen, hydroxychloroquine, azithromycin, zinc, guaifenesin, acetaminophen, meropenem, vitamin c, cefepime, albuterol, atrovent, mucomyst, dexamethasone, and methylpredisolone. Additionally, the patient received two doses of tocilizumab (*Actemra*) at 500 mg administered 12 h apart. One dose was administered during the night of day 1, and a subsequent dose was administered the following morning. Overnight, the patient remained on the non-rebreather mask with 98% saturation.

On day 2, the patient began to feel fatigued and experienced greater difficulty breathing. Because the patient had not improved on the non-rebreather mask, he was switched to BiPAP with 100% oxygen. The IL-6 levels were elevated (66 pg/mL). The acute phase reactants remained elevated: D-dimer 2027, ferritin 588.1, ESR 100 (representing an increase), and CRP > 16. AST remained elevated (55), whereas ALT was normal (40). The lymphocyte count was 1.1.

On day 3, the laboratory results revealed leukocytosis (WBC 13.1). Both AST (48) and ALT (50) remained elevated. The acute phase reactants remained high (CRP trended downward but remained elevated (14.4), ESR 102, D-dimer 2660, and ferritin 824.8 (representing an increase)). The lymphocyte counts remained low (1.12). Troponin was measured on that day and was found to be low (<0.05). The patient’s oxygen saturation remained low (88–89%), and then rose to 93% and later to 96%. The patient remained on BiPAP overnight and was then shifted to a non-rebreather mask and finally a nasal canula. 

On day 4, the patient became more stable. He reported mild shortness of breath with no acute complaints. He was taken off the BiPAP and put on the 100% non-rebreather mask. The O_2_ saturation was 99% peripherally. The acute phase reactants, although still elevated, had improved markedly from the previous day (CRP 4.7, ESR 89, D-dimer 1388, and ferritin 584.2). AST remained normal (27), and ALT was elevated (45). The lymphocyte counts were within normal limits (1.4). Leukocytosis persisted (WBC 16.7).

**Figure 1:**
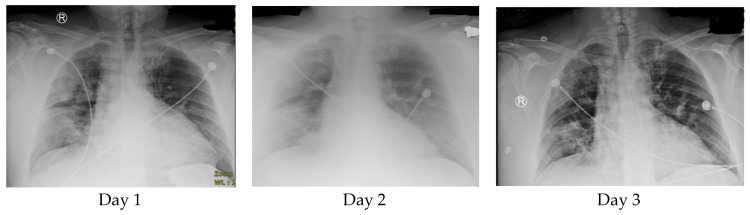
Chest X-rays on day 1, day 2, and day 5. Tocilizumab was administered as two doses of 500 mg Q12 (during the night of day 1 and the morning of day 2).

On day 5, the patient was stable and experienced only mild shortness of breath and no other complaints. He was still on a non-rebreather at 50%. The O_2_ saturation was 96%. The acute phase reactant levels remained elevated, but most values showed improvement (D-dimer 1093, ESR 40, and CRP 3.6 (representing a decrease), and ferritin 682 (representing an increase from the previous day)). The lymphocyte count was 1.3. ALT was elevated (54), and AST was normal (29). The WBC level was 14.2. A chest X-ray revealed diminished infiltrates as patchy opacities compared with imaging on day 2 (Figure 1).

On day 6, the patient remained stable with even respiration and unlabored breathing. He continued on the non-rebreather mask at 98–99% levels. All acute phase reactant levels had decreased (CRP 1.5, ESR 25, D-dimer 724, and ferritin 564.1). ALT (37) and AST (25) were both normal. Lymphocyte (3.0) and WBC (9.03) counts were in normal ranges. 

**Figure 2:**
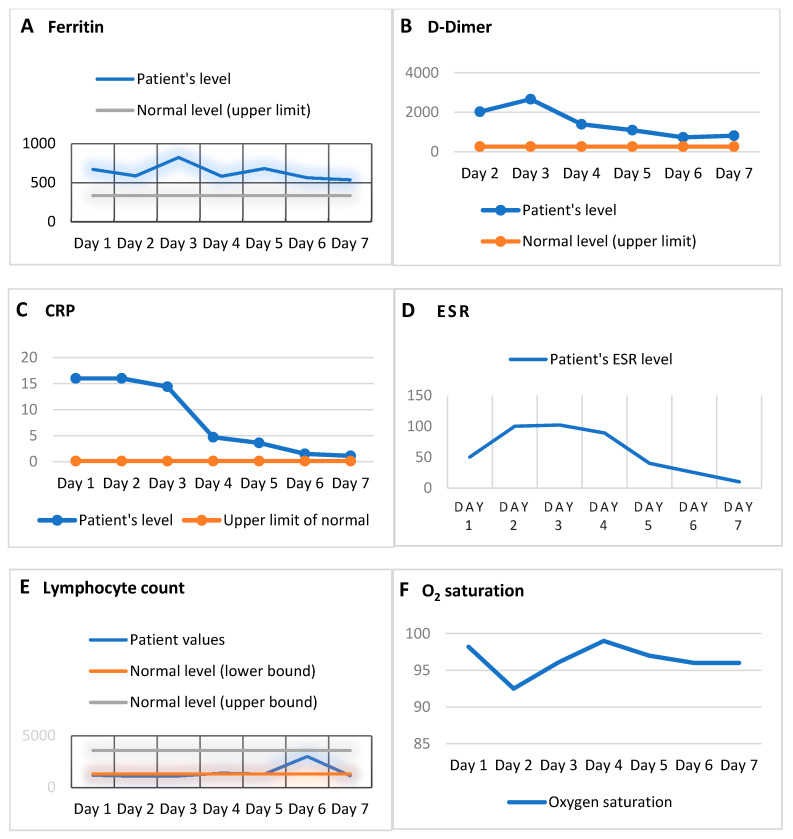
Line charts of trends in acute phase reactant biomarkers of cytokine storm—ferritin, D-dimer, and C-reactive protein (CRP) levels—along with the lymphocyte count and O_2_ saturation during the hospital stay (day 1 refers to the first day of the hospital stay). The acute phase reactants displayed an overall decline from peak levels. (**A**) Ferritin levels. (**B**) D-Dimer trends (levels first measured on day 2 of the hospital stay). (**C**) CRP levels, which were >16 for days 1 and 2 of the hospital stay. (**D**) Erythrocyte sedimentation rate (ESR) levels (reference interval 0–15). (**E**) Lymphocyte count. (**F**) Oxygen saturation (SO_2_, pulse oximetry) levels during the hospital stay. The patient was initially started on a non-rebreather mask and then switched to BiPAP on day 3.

On day 7, the patient’s condition further improved. The acute phase reactants had mostly further improved; CRP (1.1), D-dimer (811), and ferritin (538.3) remained at elevated levels, and ESR was in a normal range at 10. The lymphocyte count was 1.15. ALT remained elevated (43), and AST was normal (25). The patient continued on a non-rebreather mask and was transferred out of the ICU unit. The remainder of his hospital stay was unremarkable. He eventually fully recovered and was discharged from our hospital on day 17. The patient’s clinical progression, as shown by acute phase reactants (ferritin, D-dimer, and CRP), the lymphocyte count, ESR, and O_2_ saturation, are depicted in Figure 2.

## 3. Discussion

### 3.1. COVID-19 Pathophysiology and Cytokine Storm

Cytokine storm is seen in a variety of syndromes, including macrophage activation syndrome (MAS), hemophagocytic lymphohistiocytosis (HLH), and chimeric antigen receptor (CAR) T-cell therapy (used to treat lymphomas and leukemias) associated with hyperinflammation (known as cytokine release syndrome) [3,34,35,36,37,38]. MAS is a rare and potentially life-threatening condition entailing a cytokine storm in which macrophage and lymphocyte interactions are dysregulated, thus leading to cytokine release [39]. HLH is a critical condition marked by hyperinflammation, hemophagocytosis, and histiocyte proliferation, and is classified as either familial HLH or secondary HLH [40]. One potential mechanism for the cytokine storm in HLH disorders may be an increase in cytokines, including TNFα, IL-1, interferon gamma (IFN-γ), IL-6, sTNFRs, and soluble IL-2R (sIL-2R), as a consequence of dysregulated interactions between lymphocytes and macrophages [39]. Patients with COVID-19 exhibit clinical attributes similar to secondary HLH: hyperferritinemia, cytopenia, and ARDS [12]. 

The inflammatory cytokine storm observed in COVID-19 shares features with MAS and HLH, but has some distinguishing characteristics [3]. In COVID-19, compared with MAS, the increases in ferritin are not as high, and organ damage is primarily restricted to the lungs [38]. The clinical attributes of patients with COVID-19 also mirror those in cytokine release syndrome, which arise as a consequence of CAR-T cell therapies for certain lymphomas [20]. Importantly, not all patients with COVID-19 undergo cytokine storm; this phenomenon only occurs in a certain subset of severe cases [12]. The classic clinical picture of a patient with cytokine storm involves rapid respiratory deterioration [41,42]. Our patient began to decline and demonstrated symptoms of respiratory distress (labored breathing) on the first day of admission, and had reportedly been experiencing symptoms for 7 days prior. 

Cytokine storm instigates a robust immune-driven attack on the body, leading to ARDS (and multi-organ failure) and ultimately causing morbidity in severe COVID-19 [43,44]. The pathophysiology of cytokine storm is not fully understood, but is currently thought to be based on the mechanisms of inflammatory disorders, including MAS and cytokine release syndrome. Initially, SARS-CoV-2 binds angiotensin converting enzyme 2 (ACE-2) receptors and then invades the respiratory epithelium [37,45]. Dendritic cells and alveolar macrophages are activated because of the presence of SARS-CoV-2 and subsequently release IL-6, which is also secreted by the respiratory epithelium [37,45]. A cascade of the cytokines IL-1B, IL-12, and TNF-α results, and their secretion induces WBCs to release cytokines, thus effectively perpetuating an inflammatory cycle [12,45]. These cytokines also enter the circulation and cause systemic multi-system pathology [46]. 

The robust hyperinflammatory cytokine response induces the apoptosis of endothelial and epithelial cells in the lungs, resulting in tissue injury, leakage and edema, and ARDS [46,47]. As immune cells destroy alveolar tissue, permeability increases, thus resulting in fluid entry into the alveoli; less oxygen enters the blood, because alveolar type I cells are diminished, and a loss of surfactant leads to alveolar collapse. These responses together impair normal gas exchange [3,48]. Additionally, cytokines cause vasodilation, thereby contributing to a build-up of fluid in the alveoli, diluting surfactant, and causing alveolar collapse; as alveolar type I and alveolar type II cells are destroyed, the alveoli collapse, and ARDS ensues [48]. 

An increased synthesis of collagen and TGF-α and deposition of fibrin are also observed in the development of ARDS [3]. Features of ARDS additionally include alveolar exudate, edema, and cellular infiltration, thus resulting in damaged alveoli and limited gas exchange [21]. Xu et al. [44] have described post-mortem biopsies of COVID-19 patients with ARDS in China that showed classic ARDS-related features, such as pneumocyte desquamation, the formation of hyaline membranes, and pulmonary edema. Additionally, the presence of lymphocyte-dominated mononuclear inflammatory infiltrate in the interstitium was observed bilaterally [44]. Because of the high numbers of mononuclear lymphocytes, these T-cells have been thought to potentially enter the pulmonary circulation and trigger an inflammatory storm [21]. 

**Figure 3:**
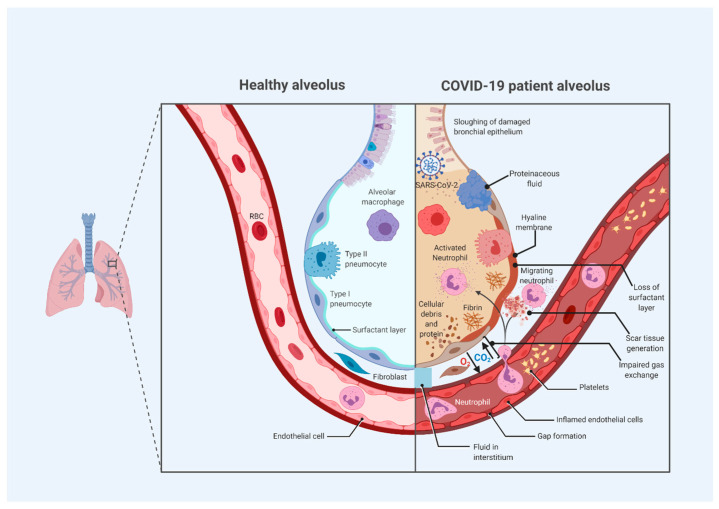
Alveolar changes due to cytokine syndrome-induced acute respiratory distress syndrome (ARDS) in severe coronavirus disease 2019 (COVID-19), according to current understanding [3,48,50,51]. This model is based in part on hypotheses (because our understanding of ARDS remains incomplete), along with reported pathological findings in COVID-19-related ARDS from post-mortem biopsies. Many of the features are hallmark characteristics of conventional ARDS. Nonetheless, COVID-19 is increasingly believed to display an atypical form of ARDS [50].

ARDS and severe illness in patients with COVID-19 usually develop 1–2 weeks after the onset of symptoms [49]. Our understanding of ARDS in COVID-19 is still evolving. It has been suggested that COVID-19 may be characterized by a unique form of ARDS, marked by a feature not seen in classic ARDS: the seemingly preserved compliance and respiratory mechanics relative to the high degree of hypoxemia [50]. Figure 3 depicts the alveolar changes that occur in severe COVID-19.

### 3.2. Diagnostic and Prognostic Roles of Serum Markers for Severe COVID-19 and Cytokine Storm

The onset of the hyperinflammation in cytokine storm may be evidenced by coagulopathy, cytopenia, tissue damage, the inflammation of liver tissue, and the activation of macrophages and hepatocytes [38]. Therefore, laboratory findings potentially suggesting the onset of cytokine storm in patients with COVID-19 may include increased levels of acute phase markers (such as D-dimer, CRP, LDH, ferritin, and troponin), low platelets, decreased fibrinogen, lymphopenia, thrombocytopenia, increased LDH, and transaminitis (elevated AST and ALT) [31,32,33,38]. Lymphopenia is a commonly reported feature of COVID-19 cytokine storm, and given that the cytokine storm must be orchestrated by other leukocytes (not T cells), an elevated WBC count is also common, suggesting that lymphopenia with leukocytosis may be a key feature in the differential diagnosis of COVID-19 [51]. In our patient, lymphopenia was observed on day 1, and on day 3 the patient developed leukocytosis. Elevated CRP and ferritin levels are key in the diagnosis of MAS and HLH. Recent studies have suggested a similar marker profile in severe COVID-19. Interestingly, cytokine storm in COVID-19 differs from that associated with other viruses as the increase in ferritin in COVID-19 is relatively modest [38]. Ferritin has been suggested as a prognostic indicator for cytokine storm in COVID-19 [38,52]. 

Serum inflammatory biomarkers may have a role in assessing disease progression, since a poor prognosis in COVID-19 appears to be correlated with abnormal serum markers and clinical attributes of cytokine storm [38]. A recent retrospective observational study of 21 patients in China comparing the attributes of moderate versus severe COVID-19 found that severe cases are more often typified by hypoalbuminemia and lymphopenia, with relatively high levels of ALT, LDH, and CRP, and particularly high levels of TNF-α, IL-2R, IL-6, and IL-10 [53]. One recent meta-analysis of 21 COVID-19-related studies on 3377 patients has shown that the levels of interleukin 10 (IL-10), IL-6, and ferritin strongly correlate with disease severity [30]. The study also showed that patients who experienced severe disease and even mortality had thrombocytopenia, lymphocytopenia, and higher WBC counts compared with those with moderate disease and disease resolution [30]. Mehta et al. [12] have suggested that inflammatory biomarkers, including ESR, decreased platelets, and increased ferritin, should be used by clinicians to risk-stratify patients who might benefit from immunomodulating treatments (such as IL-6 inhibitors). One recent study of 343 hospitalized patients with COVID-19 in Wuhan showed that D-dimer levels above 2.0 μg/mL on admission were predictive of mortality, thereby suggesting a potential prognostic role of D-dimer [54]. 

Therefore, an early assessment of these biomarkers may be critical, since rapidly identifying an emerging cytokine storm and targeting immune dysregulation in patients with COVID-19 before the rapid progression to ARDS has the potential to avoid the need for mechanical ventilation, given the low rates of survival among patients with COVID-19 who are placed on ventilators [22,38]. Experience in MAS and cytokine release syndrome has shown that intervening at an early stage of disease may be important for avoiding irreversible damage to tissue [38]. 

### 3.3. The Role of IL-6 in Cytokine Storm and COVID-19

IL-6 is a pleiotropic cytokine in the glycoprotein-130 (gp130) family of cytokines [55,56]. It has a myriad of physiological functions, including the production of acute phase reactants, stimulation of immunoglobulin production by activated B cells, regulation of bone homeostasis, lipid oxidation, glucose metabolism, and regulation of energy expenditure and appetite [56,57,58,59]. The mechanistic and multifunctional complexity of IL-6 is underscored by its anti-inflammatory effects (production of acute phase reactants from liver and epithelial cell regeneration), in addition to its pro-inflammatory properties (inflammatory cell recruitment, disrupted differentiation of regulatory T-cells, and inhibition of inflammatory cell apoptosis) [37,60]. Therefore, the role of IL-6 in COVID-19 is complex and not fully elucidated [3]. For instance, theoretically, the high IL-6 levels in COVID-19 pneumonia have been suggested to have beneficial or deleterious effects, since IL-6 in other infections can enhance viral replication or suppression in experimental models [3,61]. However, emphasis has been placed on its mainly pro-inflammatory nature in COVID-19, as confirmed by clinical reports on patients with COVID-19.

IL-6 plays a major role in various inflammatory and autoimmune disorders [62]. Importantly, an excessive generation of IL-6 during infections and tissue injury is believed to be responsible for cytokine release syndrome [59]. IL-6 dysregulation leads to the activation of complement and coagulation, inducing vascular leakage [24,63,64,65]. The activation of IL-6 is thought to be the key feature of the progression of COVID-19 pneumonia to ARDS and hyperinflammation [45].

IL-6 peak levels have been associated with pulmonary disease progression in COVID-19 [66]. Importantly, on day 2, our patient showed elevated IL-6 levels. In a recent retrospective cohort study of 201 patients in Wuhan, China, Wu et al. [67] reported a statistically significant correlation between IL-6 and mortality.

Interestingly, a role of IL-6 as a prognostic marker for COVID-19 has also been suggested. One recent meta-analysis of studies involving a total of 264 patients with COVID-19 in China reported that patients with severe COVID-19 have higher IL-6/IFN-γ ratios than those with more moderate conditions, thus suggesting that the IL-6 and IFN-γ levels may serve as a possible prognostic tool in managing patients with COVID-19 [68]. 

**Figure 4:**
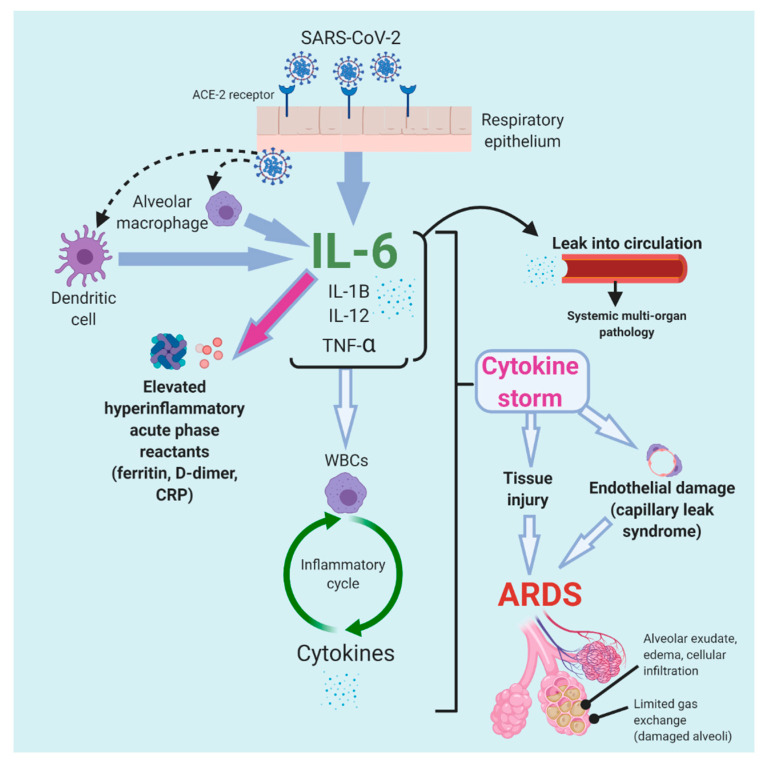
Proposed model of the cytokine storm in severe COVID-19, revealing the mechanistic complexity of the cytokine cascade and subsequent pathology [3,21,37,45,46,69]. The model is primarily based on current knowledge of cytokine storm, as seen in macrophage activation syndrome (MAS), hemophagocytic lymphohistiocytosis (HLH), and chimeric antigen receptor (CAR) T-cell therapy. Notably, cytokine storm in both MAS and HLH displays increased levels of cytokines interleukin (IL)-1, IL-2, IL-18, macrophage colony stimulating factor (M-CSF), interferon-γ, and tumor necrosis factor (TNF) α, in addition to IL-6, although definitive causality due to all these cytokines has not been determined [23].

Figure 4 illustrates the proposed inflammatory sequence of the cytokine storm observed in COVID-19 and the key role played by IL-6.

### 3.4. IL-6 Signal Transduction Pathway

IL-6 signaling occurs through a complex sequence of induction. There are two forms of the IL-6 receptor: a membrane-bound IL-6 receptor (IL-6R) and a soluble IL-6 receptor (sIL-6R) [70] IL-6 signaling primarily occurs through two modes: the classic signaling pathway mediated by membrane-bound IL-6R and the trans-signaling pathway mediated by sIL-6R [56,59]. Membrane-bound IL-6R is mainly found on hepatocytes, certain epithelial cells, megakaryocytes, and some groups of leukocytes; thus, the classic signaling pathway only occurs in these locations [55,71]. In contrast, the trans-signaling pathway can occur in all cells of the body, because it primarily requires the presence of the gp130 receptor protein, which is expressed in all cells [60].

In the classic signaling pathway, IL-6 binds membrane-bound IL-6R on the membrane of target cells and then interacts with the gp130 receptor to form a complex [55,70]. The activation of gp130 leads to the downstream activation of the Janus activated kinase-signal transducer and activator of transcription (JAK/STAT) pathway by influencing JAK (which is constitutively bound to the gp130 cytoplasmic domain), thereby inducing STAT3 phosphorylation (activation of STAT3) [56,70]. Therefore, IL-6′s interaction with its receptor ultimately leads to the activation of STAT3. The JAK/STAT3 complex translocates to the nucleus, where it activates transcription and consequently leads to the expression of acute phase proteins, which are a main feature of inflammation; their induction may have homeostatic effects [56,60].

This JAK/STAT pathway is regulated through negative feedback by suppressors of cytokine synthesis (SOCS-1 and SOCS-3) [56,59]. SOCS-1 binds activated JAK and SOCS-3 binds phosphorylated gp130, thus halting JAK activation [56]. Notably, IL-6R signaling also activates the JAK-SHP2 mitogen activated protein kinase (MAP-kinase) pathway, in addition to the JAK-STAT signaling pathway, thereby resulting in the activation of several transcription factors [56,59]. In the trans-signaling pathway, the binding of IL-6 to sIL-6R in cells that lack surface expression of the IL-6 receptor results in coupling with gp130, which in turn leads to signal transduction [56,70]. This pathway is hypothesized to be pro-inflammatory [60].

### 3.5. Potential Therapeutic Role of IL-6 Receptor Inhibition in COVID-19

IL-6 receptor inhibition has promise as a therapeutic strategy because it results in a blockade of signal transduction and gene expression [70]. IL-6 and its interactions with the IL-6 receptor stimulate the release of various acute phase proteins (including fibrinogen, CRP, hepcidin, and serum amyloid A) and promote inflammation. Therefore, the blockade of this interaction can be used to attenuate systemic inflammation in immune disorders such as Castleman disease and rheumatoid arthritis [59,72].

**Figure 5:**
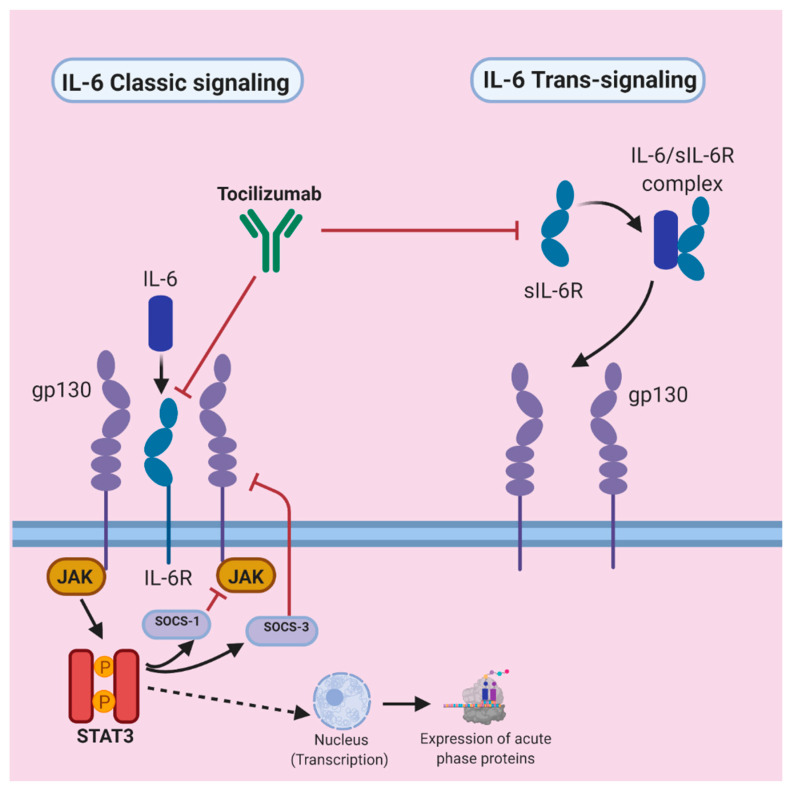
IL-6 classic and trans-signaling pathways, with a model of tocilizumab-mediated therapeutic receptor antagonism [21,55,56,60,70,76]. The diagram displays the normal sequences of the classic and trans-signaling pathways. Tocilizumab acts on both pathways (on the IL-6 receptor in the classic pathway and the soluble IL-6 receptor in the trans-signaling pathway). Note: IL-6 also uses trans presentation (in which IL-6 and membrane-bound IL-6R on dendritic cells are presented to gp130-expressing T-cells in proximity) as the third mode of signal transduction, but this pathway is not shown here [56,75].

For example, two IL-6 receptor inhibitors used to treat rheumatoid arthritis are tocilizumab and sarilumab [33]. Tocilizumab is a humanized monoclonal antibody (IgG1k subclass) that acts against both IL-6R and sIL-6R receptors and is traditionally used to treat rheumatoid arthritis, systemic juvenile idiopathic arthritis, Castleman disease, and other autoimmune disorders [37,73,74,75]. The U.S. FDA cleared tocilizumab in 2017 for the treatment of cytokine release syndrome resulting from CAR-T cell therapy [37]. Figure 5 depicts the IL-6 signaling pathway and the role of tocilizumab in IL-6 receptor antagonism.

In the U.S., tocilizumab is sold under the trade name Actemra, whereas in Europe, its trade name is RoActemra [73]. Tocilizumab has an additional use in treating the cytokine release syndrome that specifically results from CAR T-cell therapy used in B-cell malignancies [33,34]. Therefore, tocilizumab and sarilumab have been suggested to have therapeutic potential in patients with COVID-19 when there is suspicion of a cytokine storm according to elevated acute phase reactant markers (e.g., ferritin, D-dimer, CRP, and LDH), as was the case in our patient [31,32,33].

Accordingly, on day 1 of admission, we initiated an intravenous (IV) administration of tocilizumab. For the treatment for CAR-T therapy-initiated cytokine release syndrome, a conventional IV infusion of tocilizumab (alone or with corticosteroids) is indicated with a weight-based dose at 8 mg/kg for patients weighing at least 30 kg, which corresponded to a dose of 1005 mg in our patient [77]. We administered two smaller doses (500 mg each) with a 12 hour interval not based on weight, given the experimental nature of its use in COVID-19 and to mitigate any adverse effects. The National Health Commission of China’s official guidelines in the “Chinese Clinical Guidance for COVID-19 Pneumonia Diagnosis and Treatment” suggest using tocilizumab in patients who display widespread lung lesions and disease in severe stages with elevated IL-6 levels; its use is contraindicated in patients with ongoing infections, particularly tuberculosis [25]. The first dose has been suggested to be introduced at 4–8 mg/kg (but the suggested dose is 400 mg diluted to 100 mL in addition to 0.9% normal saline with more than 1 hour of infusion time) [25]. These guidelines also recommend that if patients have a suboptimal response after the first dose, a second administration with the same dose can be performed after 12 hours, with a maximum of two administrations [25]. Additionally, it has been recommend that a single dose does not exceed 800 mg [25]. Therefore, the dosing regimen for our patient was similar to this recommended dosing protocol, but with a slightly higher dose per administration. 

Importantly, IL-6 inhibitors attenuate the immune response, increasing the risk of opportunistic infections, leukopenia, and liver injury [73,78]. Some known adverse effects of tocilizumab include liver disease, allergic reactions, anaphylaxis, stomach and abdominal pain, skin and soft tissue infections, neutropenia, and hypercholesterolemia [73,79]. Reactivation of tuberculosis can occur, although this has been reported to be less common than that after treatment with tumor necrosis factor inhibitors [73]. Additionally, a possible association between tocilizumab and osteonecrosis of the jaw has been reported [80]. However, our patient did not exhibit any noticeable symptoms due to tocilizumab. Notably, IL-6 inhibitors may be cost-prohibitive [81]. Clinicians should weigh the benefits and limitations of tocilizumab therapy compared with other currently used drugs for the management of COVID-19 (Table 1). 

### 3.6. Evidence and Reports of Tocilizumab Use for COVID-19 

Our experience at a small-sized urban community hospital mirrors the positive results reported in other cases in the literature. The first report that sparked considerable attention in China described 21 patients with severe COVID-19 at two different Chinese hospitals who received one administration of tocilizumab in conjunction with standard therapy; all patients reported dramatic clinical improvements [90]. The patients received a single 400 mg tocilizumab dose and standard recommended therapy with lopinavir, methylprednisolone, oxygen treatment, and symptom relief medications. The fever declined in several days, and marked improvement was observed in all patients. Lymphocyte and CRP levels decreased after tocilizumab therapy. Fifteen patients showed a decreased oxygen intake, and one patient no longer needed oxygen therapy. Twenty patients were discharged (average of 13.5 days after tocilizumab therapy), and one was removed from the ICU [21]. No adverse effects were reported. As a result, the National Health Commission of China officially added tocilizumab to its guidelines in the “Chinese Clinical Guidance for COVID-19 Pneumonia Diagnosis and Treatment” [21,25].

Luo et al. [13] conducted a retrospective study of 15 patients with COVID-19 in Wuhan, China. The study assessed 15 patients with COVID-19 (12 males and three females with a median age of 73 years) treated with tocilizumab (eight patients were given a combination with methylprednisolone). Ten patients were administered a single dose and two were given double doses of tocilizumab. Serum acute phase reactants CRP and IL-6 were measured before and after therapy. Two patients were “moderately ill,” and the rest were in a serious or critical condition. Ten patients had at least one co-morbidity. The CRP levels decreased in all patients. The IL-6 levels decreased in most (11) patients. Mortality was seen in three patients and disease exacerbation occurred in two patients. The remaining ten patients showed stabilization. A total of four critically ill patients were given a single dose; three patients died and one experienced disease aggravation. The authors concluded that tocilizumab may have benefits. The study further suggested that a single dose of tocilizumab (even when used with glucocorticoids) may not be sufficient for improvement in critical patients, whereas repeated doses may result in improvement. The limitations of the study were its retrospective and observational nature, small sample size, and non-randomized sample, with a substantial number of patients with comorbidities. Doses of tocilizumab varied among patients, some of whom received a double dose and approximately half of whom received methylprednisolone in addition to tocilizumab. 

Other reports have described patients treated with tocilizumab with hydroxychloroquine without other major drugs, similar to our patient. De Luna et al. [91] have reported the case of a 45-year-old patient with COVID-19 in France who had sickle cell anemia with acute chest syndrome and pneumonia, and who was administered tocilizumab (in addition to hydroxychloroquine) and showed subsequent improvement. He presented on day 1 with an oxygen saturation of 91%, and then deteriorated to 80% oxygen saturation on day 2, at which time he was administered IV tocilizumab (8 mg/kg dose), in addition to the ongoing hydroxychloroquine (200 mg every 8 hours) and supplemental oxygen. On day 3, he showed improvement. In our patient, tocilizumab was administered in conjunction with ongoing hydroxychloroquine and other standard medications on day 1, followed by a second dose 12 hours later on the morning of day 2. Our patient also showed major improvement after tocilizumab administration, although we administered two doses rather than one.

Fontana et al. [92] have also described the successful use of tocilizumab in conjunction with hydroxychloroquine (with other immunosuppressive drugs) in a 61-year-old man with a previous kidney transplant for ESRD from chronic interstitial nephritis and an extensive past medical history (nodal marginal zone lymphoma, pulmonary embolism, Parkinson’s disease, and neurogenic bladder). The patient was admitted for a continual fever and shivering, and over the course of the hospital stay, was diagnosed with COVID-19. His arterial pO_2_ declined to 57 mmHg, and low-flowing oxygen via a nasal canula was started. Hydroxychloroquine (200 mg, twice per day) was administered, and the ongoing cyclosporine dose was decreased by half. Two days later, because of the lack of improvement, cyclosporine was withdrawn, and the methylprednisolone dose was increased to 16 mg daily. After 324 mg subcutaneous administration of tocilizumab, the patient’s fever resolved, the arterial pO_2_ improved progressively, and oxygen therapy was discontinued. The IL-6 levels increased 6 days after tocilizumab administration (to 619.11 pg/mL), probably because of receptor inhibition as opposed to a lack of drug efficacy. The patient was discharged on day 22 without a fever and with 95% peripheral oxygen saturation on ambient air. 

Importantly, this is the only case in the literature showing possible adverse effects of tocilizumab administration: leukopenia, neutropenia, and pseudomonas infection. However, these were treated and adequately managed. IV immunoglobulins (IVIG) were administered to alleviate ensuing leukopenia with neutropenia, which was thought to have arisen from tocilizumab administration. The patient’s leukocyte count increased. Meropenem was re-introduced after a urine culture showing multi-drug resistant *Pseudomonas aeruginosa*, which might have resulted from tocilizumab treatment, given that secondary bacterial infections are a known adverse effect. Furthermore, the patient had several comorbidities and, as a transplant patient, was already being treated with cyclosporine. Therefore, the role of tocilizumab in his outcome is difficult to determine. Additionally, because he had also been treated with hydroxychloroquine, IVIG, methylprednisolone, and azithromycin, the role of any individual drug cannot be established.

In other case reports, patients were also treated with several drugs in conjunction with tocilizumab, and they often had distinct comorbidities. Zhang et al. [93] have described the case of a 60-year-old male COVID-19 patient in Wuhan, China, with multiple myeloma who received tocilizumab and showed clinical improvement. He initially received moxifloxacin, umifenovir, and chemotherapy. He was later given methylprednisolone after experiencing chest tightness and shortness of breath. On day 9, he was intravenously administered one dose of tocilizumab. On day 12, he no longer felt chest tightness. The IL-6 levels decreased overall, with a slight momentary rebound elevation. On day 19, his CT scan showed a diminished classic ground-glass appearance. He was successfully discharged.

Other studies have reported patients who were also administered two or more doses of tocilizumab and improved. Michot et al. [94] have described the case of a 42-year-old male with metastatic sarcamatoid clear cell renal carcinoma who developed COVID-19 and showed improvement after two doses of tocilizumab. During the hospital stay, he received lopinavir-ritonavir on day 7 for 5 days. On day 8, he experienced sudden dyspnea and a decrease in oxygen saturation. The tocilizumab was administered in two doses of 8 mg/kg with an 8-hour interval in between. The patient showed clinical improvement, his fever regressed, and he had decreased oxygen requirements. His CRP levels dramatically decreased. Importantly, these positive results must be viewed by considering that the patient already had an immunosuppressed status due to cancer, and that because he received lopinavir-ritonavir, any benefit from this drug alone or the combination remains unclear. 

Cellina et al. [95] have characterized the case of 64-year-old man with no comorbidities who developed COVID-19 and experienced dyspnea and decreased oxygen saturation at 90% on day 6 of his hospital stay. On day 7, he was started on assisted ventilation, and tocilizumab was administered in two doses of 8 mg/kg, with 12 hours between doses (days 7 and 8). On day 9, the CRP and WBC count declined, and his condition improved. He was weaned off ventilatory support. On day 14, his CT revealed improvements.

Similarly, Di Giambenedetto et al. [26] have reported the successful resolution of clinical symptoms in three male hospitalized patients with COVID-19 (71, 45, and 53 years of age) after treatment with tocilizumab. Clinical improvement was seen in all three patients. In one patient (71-year-old male) who was given two doses of tocilizumab, there was a resolution of fever, the oxygen saturation improved, and the CRP returned to a normal range. In the second patient (45-year-old male) treated with two doses of tocilizumab in addition to antiviral treatment, there was clinical improvement, fever resolution, and a decrease in CRP levels after tocilizumab infusion. In the third patient (53-year-old) treated with three doses of tocilizumab, dyspnea resolved, the oxygen saturation improved, and the CRP levels decreased. 

In one investigation by Jacobs et al. [96], over the course of 24 days, 32 patients with COVID-19 were placed on extracorporeal membrane oxygen (ECMO), followed by the administration of adjunctive drugs. Five survived, and two of the five survivors were treated with tocilizumab or sarilumab. However, the clinical details of these patients and the specifics of drug administration have not been described. This study had a small sample size and was observational and retrospective. Additionally, other factors might have contributed to the outcomes (e.g., the use of ECMO).

In another observational study, Giamarellos-Bourboulis et al. [97] administered tocilizumab to six patients who were part of a group of 54 patients with COVID-19 under study for immune dysregulation and immune responses. The plasma of these patients was also studied. The absolute lymphocyte levels in the six patients decreased after tocilizumab therapy. The introduction of tocilizumab in plasma-enriched cell medium partially restored HLA-DR expression on cells. Importantly, IL-6 is believed to be the cause of decreased HLA-DR on CD14 monocytes [97]. Severe respiratory failure is associated with a significant decrease in HLA-DR expression on CD14 monocytes [97]. Therefore, the investigators suggested that tocilizumab partially relieves the immune dysregulation seen in COVID-19.

However, one patient described in the literature by Ferrey et al. [98] remained in a critical condition after tocilizumab therapy. He was a 56-year-old male with end-stage renal disease dependent on hemodialysis and with hypertension, who developed severe COVID-19. He developed ARDS and was intubated. He was given hydroxychloroquine in conjunction with standard drugs for ARDS and septic shock. On day 6, he received tocilizumab. The authors report that he remained in a critical condition at the time of the case report (his care was ongoing). Importantly, the patient had other notable comorbidities, which may have influenced his outcome.

Interestingly, the experience reported for one patient with COVID-19 by Minhai et al. [99] may even suggest a potential prophylactic role of tocilizumab in preventing severe COVID-19. The authors report the case of a 57-year-old female patient in Switzerland who experienced systemic sclerosis, insulin-dependent type 2 diabetes mellitus, and obesity, and who was already being administered tocilizumab (8 mg/kg every 4 weeks IV) before presenting to the hospital 4 weeks after the last tocilizumab dose with symptoms of COVID-19. She was quarantined at home and monitored, and she received no drug administration (her scheduled tocilizumab infusion for systemic sclerosis was postponed). Ultimately, she only developed a mild form of COVID-19 and subsequently recovered. The authors postulate that the pre-COVID-19 administration of tocilizumab might have led to a mild form of the disease and might possibly have prevented severe COVID-19, particularly given the patient’s several comorbidities, which placed her at high risk of developing severe COVID-19. Therefore, in contrast to other patients, this patient had received tocilizumab before SARS-CoV-2 infection. Nonetheless, a definitive role of tocilizumab in preventing severe COVID-19 in this patient cannot be established. Notably, she was already immunosuppressed because of her comorbidities.

The relative sudden recovery in nearly all of these reported cases after tocilizumab administration during respiratory deterioration suggests that tocilizumab may have a therapeutic benefit. Only a certain subset of patients might strongly benefit from tocilizumab. Interestingly, only one case showed any adverse effect of tocilizumab, thus suggesting the relative safety of this treatment, although potential adverse effects might still occur. Moreover, tocilizumab might have an optimal therapeutic effect when used concomitantly with other drugs. Future studies should explore which patients are most likely to benefit from tocilizumab, determine whether its sole use or combined therapy yields the most optimal effect, and identify the most effective dosing regimens. 

In addition, only one case involved a patient placed on mechanical ventilation and subsequently administered tocilizumab for successful recovery; all other patients were administered the drug at a time when their respiratory function was declining, but they had not yet been placed on mechanical ventilation, thus suggesting that tocilizumab might work most effectively at the time of emergence of a cytokine storm. Precise identification of the optimal time period in which to administer tocilizumab is critical, and further investigations may shed light on the most suitable time to use the drug. The case described by Minhai et al. [99] suggests the need for studies examining the potential role of tocilizumab in preventing severe COVID-19. A summary of existing cases in the literature that describe tocilizumab use in patients with COVID-19 is shown in Table 1. 

Our case report has some notable limitations. The patient was concomitantly treated with several drugs (including hydroxychloroquine and azithromycin) that might have influenced his clinical course. Therefore, we cannot definitely ascertain whether any one drug agent or the combination played a role in his recovery. Additionally, because most patients with SARS-CoV-2 infection recover (the case-fatality rate is low, at an estimated 2–3%), determining whether the introduction of tocilizumab might be solely responsible for lung recovery is challenging.^6^ However, given that the patient was declining with deteriorating respiratory symptoms, and that sharp recovery and relief was observed after tocilizumab administration, tocilizumab may have had a role in the patient’s clinical improvement.

## 4. Conclusions 

We present the case of a patient with COVID-19 whose condition improved after the use of tocilizumab, obviating the need for mechanical ventilation. He tolerated tocilizumab well and did not experience any known adverse effects. Our experience appears to mirror other cases in the literature that suggest a potential efficacious role of IL-6 receptor inhibition in attenuating cytokine release syndrome in patients with severe COVID-19. 

Avoiding the need for mechanical ventilation is a key therapeutic strategy in COVID-19 management. Current evidence suggests that approximately 79–86% of patients who require ventilator support experience mortality [22]. Therefore, the period of time before respiratory decline to the point at which ventilator support is needed may be especially important during the COVID-19 disease course. Therefore, although all reports to date are anecdotal and evidence is circumstantial, there may be a role for early aggressive immunosuppressive management to avert the need for ventilator support; this time period may be crucial for recovery and for preventing progression to ARDS, which can cause irreversible lung damage. 

Our case highlights the importance of the early recognition of the cytokine storm and of prompt immunosuppressive measures to halt disease progression. The early identification of clinical deterioration (as assessed by levels of cytokine storm-associated acute phase reactants) and subsequent aggressive management of patients with severe COVID-19 during the onset of respiratory decline may be key for preventing mortality. As suggested by both our experience and anecdotal cases described in the literature, acute phase reactant proteins may play an important role in the diagnosis and prognosis in severe COVID-19. Future studies are needed to determine their potential in risk stratification. According to the results of recent studies, clinicians should consider initiating measurements of IL-6 levels, the WBC count, lymphocytes, platelets, ferritin, D-dimer, CRP, and LDH for the risk stratification of cytokine storm [30,31,32,33]. 

The efficacy of combination therapy of IL-6 inhibitors with other drugs, such as hydroxychloroquine (in conjunction with azithromycin) and zinc, or other currently used COVID-19 treatments requires further investigation. Several therapies when used in concert might potentially attenuate disease pathology. We administered a smaller dose of tocilizumab than is typically used in patients with cytokine release syndrome, but slightly higher than the amounts used in previous studies. Therefore, our experience may shed light on the need for optimal therapeutic doses, which must still be established. Further investigations should explore the potential of dosages not based on weight to determine whether this approach would yield different therapeutic results. According to the literature describing cases in which variable dosing led to recovery, determining whether a single or multiple dose is most effective in COVID-19 is also needed. The optimal timing of use remains to be determined. Additionally, not all patients may be candidates for IL-6 inhibitor treatment. Decisions to administer the drug should consider co-morbidities, such as inactive tuberculosis, in conjunction with the patient’s current immune status.

Therapeutic horizons in the treatment for COVID-19 may entail various methods for preventing or disrupting disease progression in ARDS. Further investigations are warranted to shed light on alternative potential therapeutic targets for cytokine storm disruption, particularly in the IL-6 signaling pathway. For example, inhibitors of the JAK/STAT signaling pathway, which could help decrease the levels of cytokines (including IL-6 and IFN-γ) observed in severe COVID-19, have been suggested as a potential therapy [49,100]. However, to date, IL-6 receptor inhibition has garnered substantial attention in the scientific community worldwide, and the upcoming results of current trials of tocilizumab may provide a clearer picture of its efficacy. Until large randomized-controlled studies reveal conclusive evidence of the large-scale efficacy of IL-6 receptor inhibition as a viable therapeutic option in patients with severe COVID-19, clinicians should consider the anecdotal cases of the successful aversion of both cytokine storm and disease progression through the use of these drugs. Such decisions, however, must be made with caution and a consideration of the potential adverse immunomodulatory effects of IL-6 receptor inhibitor therapy. Despite the potential adverse effects of IL-6, the use of tocilizumab in conjunction with other immune-mediating medications may be a promising treatment for severe COVID-19.

Informed consent was obtained from the patient to describe and publish his case.

## Figures and Tables

**Table 1:** Comparison of tocilizumab with other drugs currently in use or under investigation for the treatment of patients with COVID-19.

Drug	General Use	Efficacy in COVID-19	Adverse Effects	Special Attributes
**Tocilizumab** **[21,73,74,79,80]**	Rheumatoid arthritis, systemic juvenile idiopathic arthritis, and polyarticular juvenile idiopathic arthritis	Anecdotal experiences suggesting a therapeutic benefit Several trials underway Large, randomized controlled studies needed	Liver disease, allergic reactions, anaphylaxis, rash, stomach and abdominal pain, skin and soft tissue infections, neutropenia, hypercholesterolemia, and possibly jaw osteonecrosis	Recombinant monoclonal antibody used for blockade of the proinflammatory cytokine IL-6 receptor Potential reactivation of tuberculosis and increased risk of infections Not usable for all patients Use potentially limited to severe COVID-19 (e.g., ICU cases) Expensive and possibly cost-prohibitive
**Hydroxychloroquine [18,82,83]**	Malaria prophylaxis and treatment, and systemic lupus erythematosus	Potential antiviral properties Demonstrated drug efficacy against *SARS in vitro* Efficacy for COVID-19 yet to be demonstrated In vivo and in vitro studies on COVID-19 currently underway	Ocular complications (corneal deposits, retinopathy), cardiotoxicity (cardiomyopathy and conduction abnormalities), cutaneous and neurologic effects, GI complications, abnormal liver function, and hepatic failure	Long half-life Increased potential for adverse effects when combined with azithromycin (which can also cause heart conduction abnormalities)
**Lopinavir-Ritonavir [14,84,85]**	HIV (lopinavir)	No benefit shown in a major recent randomized controlled trial of 199 severe COVID-19 hospitalized patients Demonstrated activity of lopinavir against coronavirus in vitro Decreased risk of ARDS or death due to SARS after treatment with lopinavir+ritonavir combined with ribavirin	GI effects (diarrhea, nausea, and vomiting), dizziness, drowsiness, headaches, hypertriglyceridemia, changes in body fat, and severe allergic reactions	Protease inhibition (lopinavir)
**Remdesivir** **[15,16,86,87,88]**	Ebola (still under investigation)	Efficacy to be determined for COVID-19 Decreased viral titers in mice infected with Middle East respiratory syndrome Phase III trial completed for use in Ebola, but no benefit shown in 2019 trial during Ebola outbreak in the Democratic Republic of Congo	Increased liver enzymes (potential liver damage), nausea, and vomiting	Disruption of viral replication by inhibiting RNA polymerase Currently in “compassionate use” for patients with COVID-19 in the U.S. and Europe
**Convalescent plasma** **[10,17,89]**	Previously used in SARS1 (2003) and MERS; also used as post-exposure prophylaxis in various infectious disease outbreaks	Limited anecdotal reports suggesting possible benefit in patients with COVID-19 Larger trials currently underway	Allergic and anaphylactic reactions, transfusion-related acute lung injury, circulatory overload (transfusion associated), infection transmission, and hemolytic transfusion reactions	Passive antibody administration
